# Herpes Simplex Virus-2 Glycoprotein Interaction with HVEM Influences Virus-Specific Recall Cellular Responses at the Mucosa

**DOI:** 10.1155/2012/284104

**Published:** 2012-05-14

**Authors:** Sarah J. Kopp, Christopher S. Storti, William J. Muller

**Affiliations:** ^1^Department of Microbiology-Immunology, Northwestern University Feinberg School of Medicine, Chicago, IL 60611, USA; ^2^Department of Pediatrics, Northwestern University Feinberg School of Medicine, Chicago, IL 60611, USA

## Abstract

Infection of susceptible cells by herpes simplex virus (HSV) requires the interaction of the HSV gD glycoprotein with one of two principal entry receptors, herpes virus entry mediator (HVEM) or nectins. HVEM naturally functions in immune signaling, and the gD-HVEM interaction alters innate signaling early after mucosal infection. We investigated whether the gD-HVEM interaction during priming changes lymphocyte recall responses in the murine intravaginal model. Mice were primed with attenuated HSV-2 expressing wild-type gD or mutant gD unable to engage HVEM and challenged 32 days later with virulent HSV-2 expressing wild-type gD. HSV-specific CD8^+^ T cells were decreased at the genital mucosa during the recall response after priming with virus unable to engage HVEM but did not differ in draining lymph nodes. CD4^+^ T cells, which are critical for entry of HSV-specific CD8^+^ T cells into mucosa in acute infection, did not differ between the two groups in either tissue. An inverse association between Foxp3^+^ CD4^+^ regulatory T cells and CD8^+^ infiltration into the mucosa was not statistically significant. CXCR3 surface expression was not significantly different among different lymphocyte subsets. We conclude that engagement of HVEM during the acute phase of HSV infection influences the antiviral CD8^+^ recall response by an unexplained mechanism.

## 1. Introduction

Herpes simplex virus type 2 (HSV-2) is a common cause of infection, with 17% of American adults seropositive [1]. HSV-2 is most commonly associated with genital infection, and spread of the virus within the population generally results from reactivation of latent infection and subsequent viral shedding [2]. 

Infection of susceptible human and mouse cells by HSV requires binding of the viral glycoprotein gD with one of its cell surface receptors [3, 4]. HSV gD binds to three general classes of surface receptors, including herpesvirus entry mediator (HVEM), nectin-1 and -2, and specific sites in heparan sulfate [3]. Of these, HVEM and nectin-1 appear to mediate viral entry most efficiently in both humans and mice [5, 6]. The, mouse receptors are orthologous to the human receptors and HSV disease in mice resembles that in humans, allowing application of mouse models to the study of HSV pathogenesis in humans.

HVEM is a member of the tumor necrosis factor (TNF) receptor superfamily of proteins [7]. HVEM is expressed in many tissues, but its principal natural function appears to be in regulating immune responses through interactions with the activating ligand LIGHT or the attenuating binding partners B and T lymphocyte attenuator (BTLA) or CD160 [8]. Although lymphocytes are not thought to be significant targets of HSV infection, since these natural HVEM ligands can either enhance or inhibit lymphocyte activation, it has been suggested that the gD-HVEM interaction could influence lymphocyte-mediated immunity to HSV. The immunologic effects of gD binding to HVEM during HSV infection have not been elucidated in detail, and although our prior work in a murine intravaginal challenge model identified differences in chemokine responses at the mucosa depending on whether virus could engage HVEM, acute cellular responses were not appreciably affected [9].

The possibility that engagement of HVEM by gD can influence memory cellular immune responses to HSV is raised by recent studies of candidate DNA vaccines encoding fusion proteins consisting of viral antigens combined with HSV gD. The results showed that protective antigen-specific CD8^+^ T-cell responses were enhanced by the presence of gD sequences, providing the domain required for binding to HVEM was functional [10–12].

Although most immune cells express HVEM, in the current study we focused on lymphocytes, including regulatory T cells (Tregs). Expression levels of HVEM on immune cells vary at different times in the immune response. Naïve and some memory CD4^+^ and CD8^+^ lymphocytes constitutively express HVEM, but expression is downregulated after activation [13, 14]. In mice, lack of HVEM or BTLA expression leads to an increased population of circulating CD8^+^ T cells with an activated memory phenotype [14]. Optimal activation of dendritic cells (DCs) by CD4^+^ memory T cells is dependent on the expression of LIGHT [15]. B cell responses may also be potentiated by HVEM/LIGHT interactions [16]. In contrast to effector lymphocytes, Tregs increase HVEM expression after activation [17]. Optimal effector responses of Tregs require both HVEM and BTLA [17]. Intriguingly, HVEM is not expressed on T cells of mice that lack Foxp3 [17], a transcription factor which defines the major Treg subset [18]. Together, these observations suggest an important role for HVEM in the generation and function of effector memory lymphocytes and in the function of Tregs.

In this study, we apply a murine intravaginal model of HSV-2 infection to investigate a role for engagement of HVEM in influencing recall immune responses. We observe differences in the HSV-specific CD8^+^ T-cell recall response at the genital mucosa based on the gD-HVEM interaction during priming. Differences in replication of the priming virus at the mucosa do not explain the observed differences in CD8^+^ T-cell infiltration, and neither CD4^+^ T cell frequency nor CXCR3 expression on responding cells differ for the two conditions. Although we observed a trend suggesting an inverse association with CD4^+^ Foxp3^+^ Tregs, we are unable to clearly implicate these cells as contributing to the different CD8^+^ T-cell recall responses we observed. We conclude that the interaction of gD and HVEM during acute infection with HSV may influence the magnitude and quality of the subsequent recall response at the genital mucosa, though the mechanism remains to be elucidated.

## 2. Materials and Methods

### 2.1. Animal Experimentation Guidelines

Animal care and use in this study were in accordance with institutional and NIH guidelines.

### 2.2. Cells and Viruses

Vero cells were cultured in Dulbecco's modification of Eagle's (DME) medium plus 10% fetal bovine serum (FBS) and 1% penicillin-streptomycin and were used for the propagation and titering of virus. Plaque titrations were performed by standard methods.

HSV-2 strain 333 was originally isolated from a genital lesion and underwent limited passage in human cells [19]. The virus was plaque-purified and passaged no more than three times in Vero cells. Modifications to the glycoprotein gD of HSV-2(333) were previously described [9]. The virulent strain HSV-2(333)/gD used for this study contains wild-type gD flanked by FRT recombination sites. Attenuated versions of HSV-2(333) were created by insertion of a *lacZ* expression cassette (which results in expression of *β*-galactosidase) in the UL3-4 intergenic region, as described previously for wild-type HSV-2(333) [20]. Two versions of attenuated virus were used: one contains the wild-type gD flanked by FRT sites (designated HSV-2(333)/gD-*β*gal) and the second contains a mutant form of gD lacking amino acids 7-15, rendering it unable to engage HVEM (designated HSV-2(333)/Δ7-15-*β*gal). Prior studies (unpublished) established that these viruses could infect mice but did not lead to mortality or significant clinical symptoms compared to infection with wild-type viruses at the inocula used. The Δ7-15 mutation introduced into the gD gene had only minor effects on mucosal replication relative to virus with wild-type gD in our prior studies [9].

### 2.3. Animal Procedures

Female C57BL/6 mice between 6–10 weeks of age were purchased from Jackson Labs and maintained in specific-pathogen-free conditions until inoculation. Mice were transferred to a containment facility for inoculation, which occurred before 12 weeks of age. Susceptibility of mice to intravaginal infection was ensured by subcutaneous injection 6 days prior to inoculation with 2.5 mg of medroxyprogesterone acetate (Depo-Provera, Pharmacia) in phosphate-buffered saline (PBS) [21]. At the time of inoculation, mice were anesthetized with ketamine-xylazine, vaginas were swabbed to clear secretions, and virus was delivered intravaginally via micropipette in 20 mL total volume. Virus was diluted in PBS containing 1% inactivated calf serum and 0.1% glucose to deliver 6 × 10^5^ PFU/mouse. All mice first received inoculation with attenuated viruses (“priming”) and were challenged with virulent HSV-2(333)/gD 32 days after priming (“challenge”). Mice were sacrificed 3 days after challenge for evaluation of cellular immune responses. Clinical symptoms were generally not observed after either inoculation, though sporadically individual mice primed with attenuated viruses had perivaginal hair loss or mild lesions which resolved prior to challenge.

### 2.4. Measurement of Viral Replication in the Vaginal Tract

To assess replication of the attenuated virus in the vaginal tract, secretions were collected from infected mice at indicated times. Sterile PBS was instilled intravaginally in a 40 *μ*L volume and pipetted in and out 2-3 times (see e.g., [22]). This was repeated twice and samples pooled for analysis by standard plaque assay. Samples were stored at −70°C until analysis.

### 2.5. Isolation of Lymphocytes

Lymphocytes were isolated from draining lymph nodes (DLN) and vaginal tissue. DLNs from each mouse were separately homogenized in RPMI-1640 with 2% heat-inactivated FBS. Red blood cells were lysed with ACK buffer, and cells were washed, counted, and maintained in complete medium (RPMI-1640, 10% FBS, 1 mM sodium pyruvate, 0.1 mM nonessential amino acids, 1% penicillin-streptomycin, and 20 mM *β*-mercaptoethanol) prior to labeling for flow cytometric evaluation.

Vaginal tissue was processed by a similar protocol we previously used [9], based on method described by Gierynska et al. [23]. Briefly, isolated tissue was washed in Hanks balanced salt solution (HBSS), cut into small pieces, and incubated in HBSS with collagenase D (1 mg/mL) for 1 hour at 37°C with gentle agitation. Digested tissue was pressed through a cell strainer, washed in RPMI-1640 with 2% heat-inactivated FBS, and the cells counted and resuspended in complete medium. There were no differences between the groups in the overall numbers of cells recovered from any tissue.

### 2.6. Measurement of Murine T-Cell Responses by Flow Cytometry

Isolated cells were washed and resuspended in FACS buffer. Surface labeling included fluorescently conjugated antibodies to murine CD4, CD8, CXCR3, and DimerX-PE reagent (BD Biosciences) preloaded with the gB_496–503_ peptide according to the manufacturer's instructions. A cell permeabilization kit was used for intracellular labeling with fluorescently conjugated antibody to murine Foxp3 (BD Biosciences). Cells were analyzed using an LSR II flow cytometer (Becton-Dickinson) and FloJo software (TreeStar). Lymphocytes were gated based on forward and side-scatter characteristics, with isotype control antibodies used to determine the threshold for positive labeling with Foxp3 and CXCR3. Lymphocytes from uninfected mice were used for negative control labeling with the DimerX reagent. A minimum of 10000 events were collected for each condition analyzed. Percentages in different subsets were measured based on fluorescence detected at relevant wavelengths.

### 2.7. Statistical Tests

Mean percentages of fluorescently labeled cells within a population were compared between experimental groups using the unpaired Student's *t*-test.

## 3. Results

### 3.1. Replication of the Virus within the Murine Vaginal Tract Is Only Minimally Affected by the Ability of the Virus to Interact with HVEM

**Figure 1:**
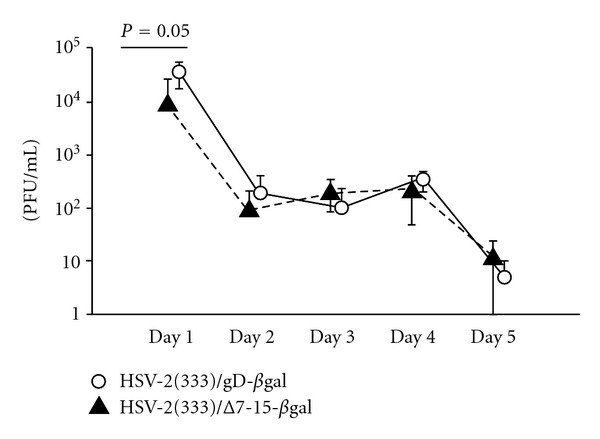
HSV-2 titers in the vaginal tract after infection with the attenuated viruses HSV-2(333)/gD-*β*gal or HSV-2(333)/Δ7-15-*β*gal are only minimally affected by engagement of HVEM. Viral titers were measured in vaginal secretions on the days indicated after virus inoculation. Five mice per group were inoculated intravaginally with 6 × 10^5^ PFU of HSV-2(333)/gD-*β*gal (open circles) or HSV-2(333)/Δ7-15-*β*gal (filled triangles). Symbols denote mean titers (±standard deviation) for each group. There was a statistically significant difference (*P* = 0.05) in mean titer on day 1, with approximately 0.5 log lower titer of HSV-2(333)/Δ7-15-*β*gal at this time point. Measured titers were statistically equivalent at subsequent time points.

Our prior studies had noted only minor differences in viral replication in the vaginal tract after infection with the virulent strains HSV-2(333)/gD and HSV-2(333)/Δ7-15 [9]. To confirm that the attenuated strains of these viruses (HSV-2(333)/gD-*β*gal and HSV-2(333)/Δ7-15-*β*gal, resp.) also had similar replication kinetics in the vaginal tract, we inoculated groups of mice with the two attenuated viruses and measured viral titers in vaginal secretions over time (Figure 1). The strain of virus unable to engage HVEM was about 0.5 log lower in titer on day 1 (*P* = 0.05), but there were no statistical differences in titer on subsequent days. We conclude that the Δ7-15 deletion in gD, which abrogates the ability of the virus to use HVEM as an entry receptor without altering its ability to use nectin-1, does not greatly alter *in vivo* replication of the attenuated virus strains.

### 3.2. HSV-Specific CD8^+^ T-Cell Mucosal Recall Responses Are Diminished by HSV-2 Interaction with HVEM during Priming

**Figure 2:**
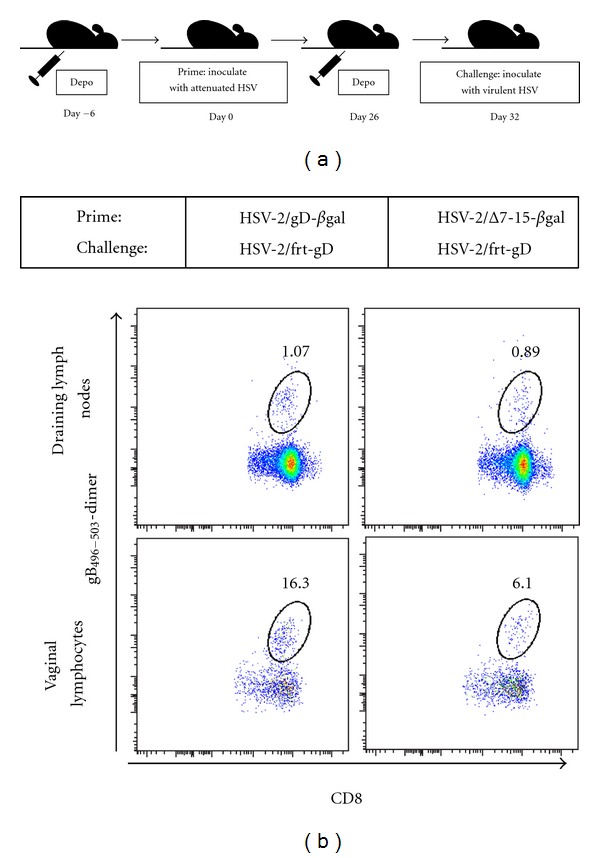
(a) Schematic of experimental design. (b) The HSV-specific CD8^+^ T cell response at the vaginal mucosa in individual mice differs in the recall phase depending on whether HVEM was engaged during priming. Representative examples from individual mice within each condition analyzed using identical gating strategies are shown.

**Figure 3:**
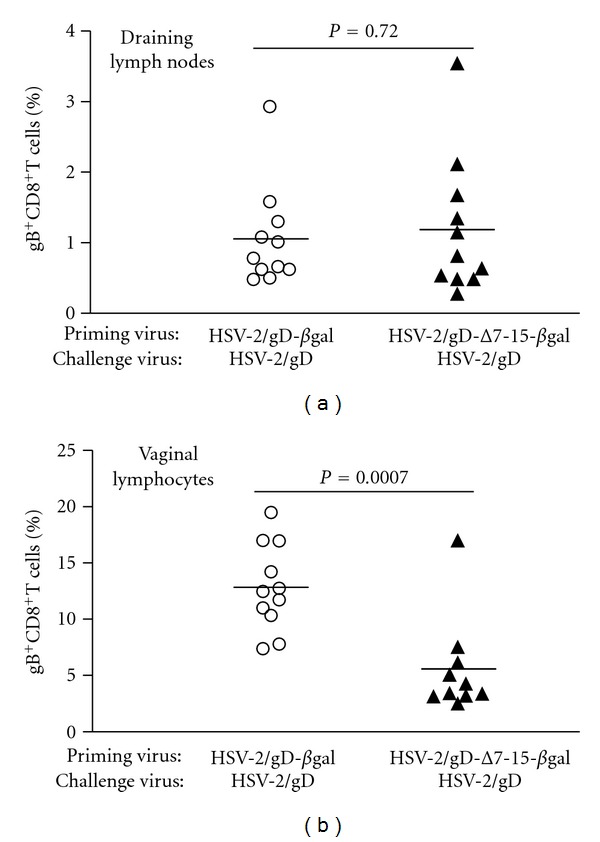
HSV-specific CD8^+^ T cell frequencies do not differ in lymphocytes isolated from (a) draining lymph nodes from groups of mice primed with viruses than can (HSV-2/gD-*β*gal, left, open circles) or cannot (HSV-2/gD-Δ7-15-*β*gal, right, filled triangles) engage HVEM, but are significantly different at the (b) vaginal mucosa. Symbols show data from individual mice, with the horizontal line designating the mean for each experimental group. Data are pooled from three independent experiments with 3-4 mice per group.

Female mice were primed with either HSV-2(333)/gD-*β*gal or HSV-2(333)/Δ7-15-*β*gal and challenged with HSV-2(333)/gD (Figure 2(a)). Studies of the CD8^+^ T-cell immune response after HSV infection of C57BL/6 mice have identified an immunodominant H-2K^b^-restricted epitope in the gB glycoprotein (gB_496–503_), which accounts for >70% of the CD8^+^ cellular immune response [24, 25], allowing measurement of HSV-specific responses using fluorescently labeled MHC dimers loaded with the dominant peptide [26]. HSV-specific CD8^+^ lymphocytes were present at a significantly greater frequency in vaginal mucosa three days after challenge with HSV-2(333)/gD in animals which were previously primed with HSV-2(333)/gD-*β*gal, compared to those primed with HSV-2(333)/Δ7-15-*β*gal (Figures 2(b) and 3). 

Priming with virus able to engage HVEM led to more than twofold increase in the mean percentage of HSV-specific CD8^+^ T cells recovered at the vaginal mucosa compared to priming with virus unable to engage HVEM. No differences were seen in the frequency of HSV-specific CD8^+^ lymphocytes isolated from DLN (Figures 2(b) and 3). There were also no differences found in the mean percentage of CD8^+^ T cells (independent of gB_496–503_ antigen specificity) isolated from vaginal mucosa or DLN between the two groups (data not shown). We conclude that the interaction of HSV gD with HVEM during initial infection of C57BL/6 mice results in an increased frequency of HSV-specific CD8^+^ T cells at the mucosa but not the DLN during the recall response after challenge with virulent HSV-2 expressing wild-type gD. 

### 3.3. Engagement of HVEM during Priming Does Not Alter CD4^+^ T-Cell Frequencies in the DLN or Vaginal Mucosa during the Recall Response

**Figure 4:**
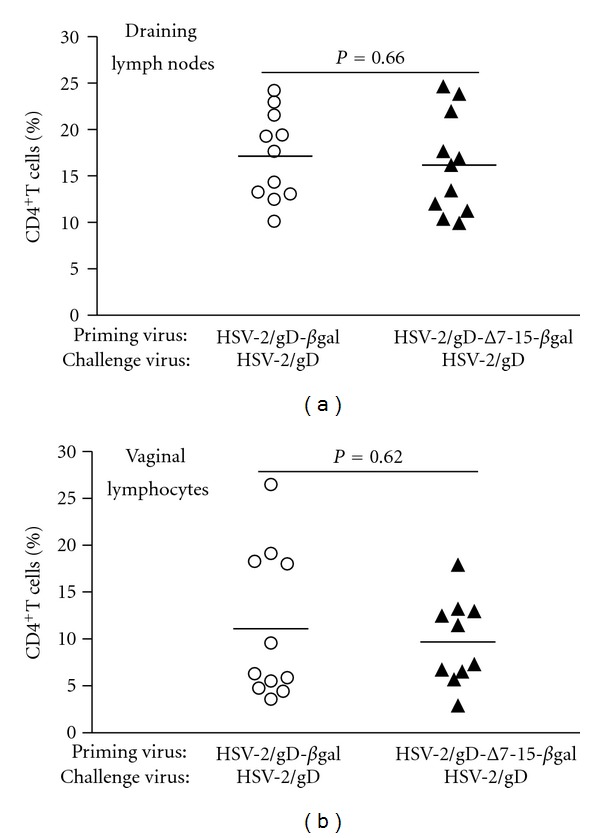
CD4^+^ T cell frequencies do not differ in draining lymph nodes (a) or vaginal mucosa (b) among lymphocytes isolated from groups of mice primed with viruses that can (HSV-2/gD-*β*gal, left, open circles) or cannot (HSV-2/gD-Δ7-15-*β*gal, right, filled triangles) engage HVEM. Symbols show data from individual mice, with the horizontal line designating the mean for each experimental group. Data are pooled from three independent experiments with 3-4 mice per group.

Prior investigators have demonstrated a crucial role for CD4^+^ T cells to direct entry of virus-specific CD8^+^ T cells into mucosal tissue after acute HSV infection via secretion of interferon-*γ* (IFN-*γ*) and induction of chemokine production [27]. Our previous study detected no differences in the frequencies of IFN-*γ* producing CD4^+^ T cells after acute infection using viruses that were or were not able to engage HVEM [9]. Since differences in the frequencies of CD4^+^ T cells (and by inference the amount of IFN-*γ* produced) in the vaginal tissue during the recall response could explain differences in the frequencies of infiltrating virus-specific CD8^+^ T cells, we measured CD4^+^ T-cell infiltration into mucosal tissue and DLN during the recall response (Figure 4). In contrast to the results for HSV-specific CD8^+^ T cells, no differences in the frequencies of infiltrating CD4^+^ T cells were seen in the vaginal mucosa or DLN of mice primed with HSV-2(333)/Δ7-15-*β*gal compared to those primed with HSV-2(333)/gD-*β*gal. We conclude that the initial interaction of HSV with gD during the priming phase of the immune response in C57BL/6 mice does not alter the CD4^+^ T-cell response in DLN or vaginal mucosa during the challenge phase. However, our data cannot rule out differences in IFN-*γ* production by responding CD4^+^ T cells in the recall response. 

### 3.4. Regulatory T-Cell Infiltration into Infected Tissue during the Recall Response May Be Altered by Prior Engagement of HVEM

**Figure 5:**
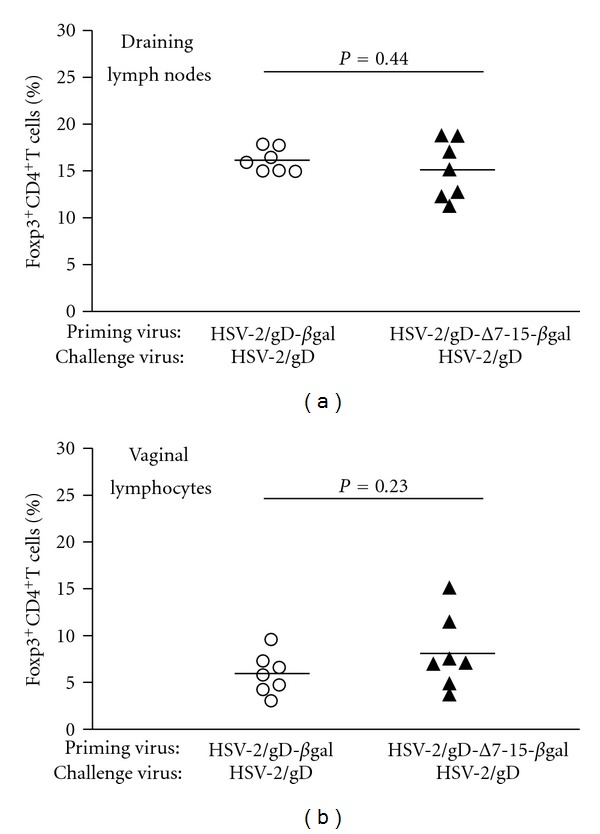
Foxp3^+^CD4^+^T cell frequencies do not significantly differ in draining lymph nodes (a) or vaginal mucosa (b) among lymphocytes isolated from groups of mice primed with viruses that can (HSV-2/gD-*β*gal, left, open circles) or cannot (HSV-2/gD-Δ7-15-*β*gal, right, filled triangles) engage HVEM. Symbols show data from individual mice, with the horizontal line designating the mean for each experimental group. Data are pooled from three independent experiments with 3-4 mice per group.

Tregs have an important role in directing the acute cellular immune response to mucosal HSV infection in mice by altering the chemokine and cytokine gradient between the infected tissue and draining lymph nodes [28]. Since alterations in Treg frequencies in different tissues during the recall response could lead to the differences we observed in antiviral CD8^+^ T cell frequencies, we assessed Treg frequency during the recall response in our infection model. Foxp3^+^ CD4^+^ Tregs were detected at a lower frequency in vaginal mucosa of mice primed with HSV-2(333)/Δ7-15-*β*gal compared to mice primed with HSV-2(333)/gD-*β*gal (Figure 5); however, this difference did not reach statistical significance. No differences were detected in Foxp3^+^CD4^+^ Tregs in DLN between the different experimental groups. Although the trend observed for Treg infiltration into vaginal mucosa during the recall response was inversely related to the infiltration of HSV-specific CD8^+^ T cells, due to the lack of statistical significance we are unable to firmly implicate a role for Tregs in directing this response.

### 3.5. Homing Receptor Expression on HSV-Specific T Cells during the Recall Response Is Not Affected by Prior Engagement of HVEM

**Figure 6:**
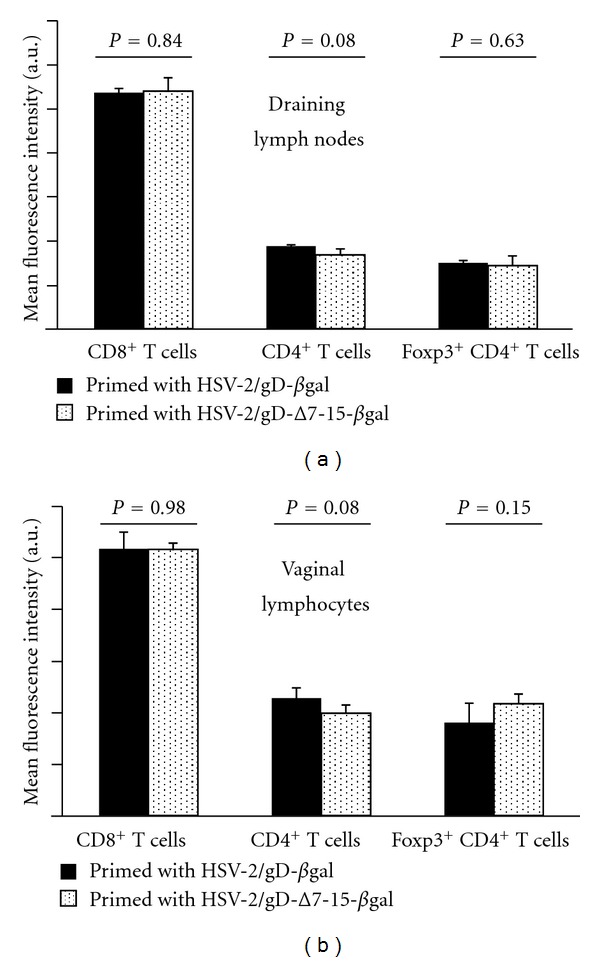
CXCR3 expression on CD8^+^, CD4^+^, and Foxp3^+^ CD4^+^ T cells does not statistically differ between groups of mice primed with viruses that can (HSV-2/gD-*β*gal, black bars) or cannot (HSV-2/gD-Δ7-15-*β*gal, stippled bars) engage HVEM. Gates were chosen to exclude nonspecifically labeled cells based on isotype control antibody staining. Data are expressed as mean fluorescence intensity (MFI) of the indicated cell population from 3-4 mice per condition, plus the standard deviation. *P* values reflect differences in mean CXCR3 MFI for each cell population between groups of mice primed with the different viruses.

Recruitment of effector T cells to infected mucosal tissue is dependent on expression of CXCR3 [27], which is the receptor for the chemokines CXCL9 and CXCL10. We previously observed that levels of both CXCL9 and CXCL10 were affected in the acute phase of HSV infection based on whether the virus could engage HVEM [9]. CXCR3 is important in priming T cell responses and has a potential role in inducing T cell memory [29], and a possible explanation for the differences in CD8^+^ T cell infiltration we observed could be related to expression of this homing receptor. Accordingly, we assayed the expression of CXCR3 on different subsets of lymphocytes in draining lymph nodes and vaginal tissue during the recall response. Mean fluorescence intensity of CXCR3 expression on CD8^+^, CD4^+^, and Foxp3^+^CD4^+^T cells did not differ based on whether the priming virus was able to engage HVEM (Figure 6). We conclude that despite our prior observation of differences in chemokine production based on HVEM expression in the acute response [9], no measurable differences in chemokine receptor expression may be demonstrated during the recall response on the basis of gD interactions with HVEM during priming. 

## 4. Discussion

Our study describes a role for the acute interaction of HSV-2 gD with HVEM in modifying the antiviral recall immune response after intravaginal infection of mice. Our principal observation was that engagement of HVEM during acute infection (priming) increased the HSV-specific CD8^+^ T-cell response at the mucosa during rechallenge. We investigated several possible contributing factors for this observation, but were unable to clearly demonstrate roles for CD4^+^ T-cells, Foxp3^+^CD4^+^ Tregs, or expression of the chemokine receptor CXCR3 on responding cells in relation to whether the priming virus was able to engage HVEM.

We used the well-established murine intravaginal challenge model for these studies [21] to investigate the antiviral cellular recall response at the mucosa. This model is limited for studying HSV-2 pathogenesis in mice by mortality in productively infected animals when wild-type virus is used, requiring us to generate initial antiviral immune responses by priming with an attenuated virus. Mice can be protected against disease after intravaginal challenge with wild-type virus by a variety of means, including passive antibody transfer [30] and by generation of HSV-specific cellular responses [23, 31, 32], and from preliminary studies we knew that our attenuated viruses could provide protection against subsequent wild-type HSV challenge. We did not formally demonstrate the mechanism for this protection, but we expect that priming with either virus leads to both humoral and cellular immunity capable of mediating protection. Since our prior work [9] and current data (Figure 1) also demonstrate that mutation of gD to abrogate interaction with HVEM has only minor effects on local viral replication in the intravaginal model, we believe our results reflect an effect that is primarily attributable to the relative ability of the priming viruses to engage HVEM. Memory T cell responses are generated from a minor population within the effector pool of CD8^+^ T cells [33], and antiviral T cell activation and expansion are barely underway 24 hours after infection [34]. Also, the numbers of memory T cells generated following resolution of an acute infection are thought to depend at least in part on the peak response during the effector phase of the cellular immune response [34]; our prior data did not identify differences in the peak HSV-specific CD8^+^ T cell response on the basis of viral engagement of HVEM [9]. Therefore, the small replication difference between the two viruses one day after inoculation (0.5 log PFU/mL) would be unlikely to explain the more than twofold difference in recall response at the mucosa.

A second potential alternative explanation for our findings is that the gB-specific recall response is specifically altered by engagement of HVEM during priming, leading to a recall response that is perhaps HSV-specific but involves subdominant epitopes. Consistent with this possibility is our observation that overall CD8^+^ T cell frequencies in mucosal tissue were unaltered in the recall response. However, our prior findings of no differences in the acute CD8^+^ T cell response to gB_496–503_ after challenge with virulent HSV-2(333)/gD compared to HSV-2(333)/Δ7-15, combined with the understanding that memory responses are partly dependent on peak responses [34], suggest against this possibility. Nevertheless, we are unable to completely rule out this alternative explanation with our data.

HVEM is a member of the TNF receptor family, which is broadly expressed in hematopoietic cells [35, 36]. Signaling through HVEM results in different responses in immune cells depending on the context in which it is engaged [8]. Engagement of HVEM by LIGHT or lymphotoxin-*α* increases T-cell activation [35], while BTLA [37, 38] and CD160 [39] attenuate T-cell activation and proliferation upon interaction with HVEM. Although lymphocytes are not generally considered to be targets of infection with HSV *in vivo*, the interaction of HSV gD on the viral envelope and on infected cells may modulate lymphocyte activity through an interaction with HVEM. HSV gD binds to HVEM in the same membrane-distal cysteine-rich domain (CRD1) [40] as both BTLA [41] and CD160 [39], and soluble gD competitively inhibits the BTLA-HVEM interaction [38]. The interaction of gD with HVEM can itself trigger NF-*κ*B activation [42]. Also, gD might competitively inhibit the binding of HVEM to BTLA or CD160, with consequences that depend on the effects of the HVEM-BTLA/CD160 interactions. The HSV gD interactions with HVEM may also alter responses of other (nonlymphocyte) immune cells which express HVEM or its ligands, such as dendritic cells, whose homeostasis is dependent on HVEM and BTLA signaling [43], and NK cells, which may be activated by engagement of CD160 [44].

Given the multiple combinations for binding between HVEM and its multiple ligands, between LIGHT and its binding partners HVEM and the lymphotoxin-*β* receptor (LT-*β*R), and the differential regulation of expression of these molecules on different cell types during an inflammatory response, a mechanism by which any of the above interactions would be altered by gD to affect memory T-cell responses is not immediately obvious. Prior studies in BTLA-deficient mice show increased differentiation of naïve CD8^+^ T cells into central memory cells in the absence of BTLA [14], suggesting that our results could be explained by interference of HVEM-BTLA signaling by gD during acute infection. Optimal Treg responses are also dependent on the HVEM-BTLA signaling pathway [17]; upregulation of HVEM by Tregs and BTLA by effector T cells after TCR stimulation suggests the hypothesis that the level of Treg signaling to effector T cells through HVEM-BTLA during the acute response may program subsequent memory cell differentiation, controlling either the numbers or other characteristics of the memory cell population (e.g., migratory characteristics might be altered by effects on chemokine receptor expression). However, as other cell types also express HVEM and BTLA, including DCs and NK cells, a role for HVEM signaling by these cells in the shaping of the memory immune response is also possible.

Several lines of evidence suggest possible ways that the chemokine and cytokine environment within which the acute immune response is developing may influence the generation and persistence of memory cells. Antigen-specific CD4^+^ T cells have recently been shown to require expression of both LIGHT and HVEM to persist as memory cells [45]. Expression of the chemokine receptor CXCR3 on T cells in DLNs has also been implicated in induction of T-cell memory [29]. Any modulation of HVEM-LIGHT signaling by gD could affect either or both of these pathways. It is also possible that memory cell generation and persistence is not affected by the initial gD-HVEM interaction, but subsequent chemokine production or chemokine receptor expression by memory cells is programmed in some manner by the initial context of HVEM signaling, leading to changes in chemokine gradients during the recall response, which change the relative infiltration of different memory lymphocytes. We did not measure the chemokine response in the challenge phase in these experiments, and CXCR3 measurements did not reveal a role for expression of this receptor in the response we observed. Further work on defining the underlying mechanism for our observations is ongoing, including evaluation of different time points and experiments using adoptive transfer and HVEM knockout mice.

To our knowledge, this study is the first to show an influence of the HSV gD-HVEM interaction on recall immune responses in an HSV infection model. However, an influence of the gD-HVEM interaction on memory immunity is not entirely unexpected based on prior vaccine studies [10–12]. In these investigations, candidate vaccines were constructed to express different viral antigens fused to the C-terminal domain of HSV-1 gD and delivered intramuscularly to mice. These constructs induced stronger immune responses than those which lacked either the gD fusion or in which gD was unable to interact with HVEM. The authors attributed this observation primarily to interference by gD with coinhibitory signaling by the natural BTLA-HVEM interaction.

Among the many questions left unanswered by our work is whether any advantage is conferred to the virus by manipulation of HVEM signaling pathways. It seems counterintuitive that HSV evolved to use a receptor that ultimately leads to a stronger recall response at the site of initial infection than if a different entry receptor had been used. One possibility is that initial establishment of infection is favored by the use of gD to disrupt HVEM signaling [9]. If virus is able to reactivate and shed even in the presence of a strong recall response, it is possible that there is no significant selection pressure against this effect. Further investigation into the pleiotropic functions of HVEM in immunity may shed light on this question.

Finally, it is worth commenting further on the implications of this observation on pathogenesis of HSV disease and possible therapeutics, including vaccination. Studies of human trigeminal ganglia and skin biopsy samples strongly support the concept that memory CD8^+^ T-cell responses are critical for the control of recurrent infection [46, 47]. Manipulation of HVEM signaling to properly direct these responses to relevant tissues could benefit therapeutic vaccine strategies [11]. There may also be implications for disease recurrence. Although a prior study of individuals with HSV-specific cellular immunity but no serologic or clinical evidence of infection failed to identify HVEM polymorphisms which altered viral entry into cells [48], it is possible that HVEM variants may lead to signaling differences that either promote or diminish effective mucosal cellular immune responses during viral reactivation. A similar survey of HVEM variants in patients with frequent recurrences has not been completed.

## 5. Conclusions

We have described a role for the initial interaction of HSV gD with HVEM in shaping the antiviral CD8^+^ T cell recall response at the mucosa. Further studies are needed to elucidate the mechanism behind this effect, and how it may contribute to HSV pathogenesis and perhaps influence the design of therapeutic interventions, including vaccines.
